# Cytoprotective Effects of Grape Seed Extract on Human Gingival Fibroblasts in Relation to Its Antioxidant Potential

**DOI:** 10.1371/journal.pone.0134704

**Published:** 2015-08-10

**Authors:** Yusuke Katsuda, Yoshimi Niwano, Takuji Nakashima, Takayuki Mokudai, Keisuke Nakamura, Satomi Oizumi, Taro Kanno, Hiroyasu Kanetaka, Hiroshi Egusa

**Affiliations:** 1 Tohoku University Graduate School of Dentistry, Sendai, Japan; 2 Kiasato Institute for Life Sciences, Kitasato University, 5-9-1 Shirokane, Minato-ku, Tokyo, Japan; University of Dhaka, BANGLADESH

## Abstract

Cytoprotective effects of short-term treatment with grape seed extract (GSE) upon human gingival fibroblasts (hGFs) were evaluated in relation to its antioxidant properties and compared with those of a water-soluble analog of vitamin E: trolox (Tx). GSE and Tx showed comparable antioxidant potential *in vitro* against di(phenyl)-(2,4,6-trinitrophenyl)iminoazanium (DPPH; a stable radical), hydroxyl radical (^•^OH), singlet oxygen (^1^O_2_), and hydrogen peroxide (H_2_O_2_). Pretreatment or concomitant treatment with GSE for 1 min protected hGFs from oxidative stressors, including H_2_O_2_, acid-electrolyzed water (AEW), and ^1^O_2_, and attenuated the intracellular formation of reactive oxygen species induced by H_2_O_2_ and AEW. Tx also reduced the H_2_O_2_- and AEW-induced intracellular formation of reactive oxygen species, but showed no cytoprotective effects on hGFs exposed to H_2_O_2_, AEW, or ^1^O_2_. These results suggest that the cytoprotective effects of GSE are likely exerted independently of its antioxidant potential.

## Introduction

Grape seed is one of the richest sources of proanthocyanidin [1,2], a polymer of flavan-3-ols with an average degree of polymerization between 2 and 17) [3,4]. Grape seed extract GSE) is noteworthy for its anti-oxidative activity including scavenging free radicals [5,6]. Besides the anti-oxidative property, it is suggested that GSE has anti-inflammatory, anti-diabetic, anti-obesity, anti-carcinogenic and anti-ageing effects [7–14].

Periodontal diseases (gingivitis and periodontitis) are chronic inflammatory diseases, which are generally caused by gram-negative bacteria, and feature gingival inflammation. Lipopolysaccharide is a cell wall component of gram-negative bacteria, which inhabit in almost all the subgingival tissues, and acts as pathogenic and exacerbating factors for periodontal diseases through inflammatory response [15–17]. One of the main targets of LPS is human gingival fibroblasts (hGFs) that play a pivotal role in inducing periodontal tissues injury through cytokine production such as IL-6 and IL-8 [18–20].

Previously, we revealed that pretreatment of human gingival fibroblasts (hGFs) with grape seed extract (GSE) containing proanthocyanidin for 1 min elicited cytoprotective effects upon hGFs exposed to harsh environmental conditions; short-term exposure of hGFs in the mitotic phase to pure water or physiologic saline resulted in the low recovery of viable cells [21]. GSE pretreatment improved the recovery of cells exposed to pure water or physiologic saline. In addition, hGFs exposed to GSE for 1 min were proliferous, even after culture in a serum-free medium. In that study, it was also shown that intracellular formation of reactive oxygen species (ROS) induced by culture in serum-free medium was inhibited in cells pretreated with GSE for 1 min. Those results suggested that, because of its cytoprotective effects, GSE could be a novel prophylactic and/or therapeutic agent for oral injury.

Aside from our previous study, several studies have shown that polyphenols (including proanthocyanidin) can protect cells. For instance, the reduced cell viability and oxidative stress in HepG2 cells induced by tert-butyl hydroperoxide can be mitigated by treatment with proanthocyanidin for 6 h [22]. Also, ellagic acid has been shown to ameliorate the cytotoxic effect of paraquat (1,1′-dimethyl-4,4′-bipyridinium dichloride) on human alveolar A549 cells *via* its antioxidant action [23]. In addition, epigallocatechin-3-gallate shows a cytoprotective effect on mycotoxin-induced cytotoxicity in the human colon adenocarcinoma cell line HT29 through anti-oxidative and anti-inflammatory mechanisms [24]. Also, lemon grass (*Cymbopogon citratus* Stapf) polyphenols can protect human umbilical vein endothelial cells from oxidative damage induced by high glucose, hydrogen peroxide (H_2_O_2_), and oxidized low-density lipoprotein [25]. Such studies suggest that cytoprotective effects are exerted *via* the antioxidant action of those polyphenols.

Major differences between the studies described above and our previous study were that the: (i) duration of GSE treatment was as short as 1 min; (ii) involvement of an antioxidant action in the cytoprotective effects of GSE was not apparent. Hence, we examined further the cytoprotective effects of short-term GSE treatments on hGFs exposed to various oxidative stressors in relation to the antioxidant properties of GSE *in vitro*. We also compared the effects of GSE with those of a water-soluble analog of vitamin E: trolox (Tx).

## Materials and Methods

### Test materials and reagents

GSE (Leucoselect) was obtained from Indena (Milan, Italy). Di(phenyl)-(2,4,6-trinitrophenyl)iminoazanium (DPPH) was purchased from Tokyo Chemical Industry (Tokyo, Japan). 5,5-Dimethyl-1-pyrroline *N*-oxide (DMPO), and xanthine oxidase (XOD) were from Labotec (Tokyo, Japan). 2,2,5,5-Tetramethyl-3-pyrroline-3-carboxamide (TPC), 4-hydroxy-2,2,6,6-tetramethylpiperidine-1-oxyl (TEMPOL), hypoxanthine (HPX), superoxide dismutase (SOD; from bovine erythrocytes), and allopurinol were obtained from Sigma-Aldrich (Saint Louis, MO, USA). 6-Hydroxy-2,5,7,8-tetramethylchroman-2-carboxylic acid (Tx), rose bengal, and sodium azide (NaN_3_) were purchased from Wako Pure Chemical Industries (Osaka, Japan). All other reagents used were of analytical grade.

### Assay for polyphenols in GSE and liquid chromatography/mass spectrometry (LC/MS) analyses of GSE

Total polyphenol content was determined by the Folin–Denis method [26]. In brief, 3.2 mL of pure water, 200 μL of 1 mg/mL of GSE, 200 μL of Folin & Ciocalteu’s Phenol Reagent and 400 μL of saturated sodium carbonate solution were mixed. Absorbance was determined at 760 nm using a Microplate Reader (FilterMax F5; Molecular Devices, Sunnyvale, CA, USA) after standing for 30 min. Freshly prepared gallic acid was used as a standard.

For LC/MS analyses, GSE was dissolved in pure water to make a concentration of 1 mg/mL followed by passage through a filter (polyvinylidene difluoride; pore size, 0.2 μm). The resultant sample was injected into the electrospray ion source of a QSTAR Elite electrospray ionization (ESI) quadruple time-of-flight mass spectrometer (AB Sciex; Framingham, MA, USA) coupled to Agilent 1200 series (Agilent Technologies, Santa Clara, CA, USA). Chromatographic separation was undertaken on an Inertsil ODS-4 (3.0 × 250 mm, GL Sciences, Tokyo, Japan) at 40°C. With regard to gradient elution, solvent A was water with 2 mM ammonium acetate, and B was methanol with 2 mM ammonium acetate. Gradient elution was 0–30 min and 5–100% B. Flow rate was 0.5 mL/min, the injection volume was 5 μL, and UV detection was carried out by a photodiode array detector. Electrospray ionization-mass spectrometry was recorded for 30 min in the *m/z* region from 100 to 2000 Da with the following instrument parameters: ion spray voltage = 5500 V, source gas = 50 L/min, curtain gas = 30 L/min, declustering potential = 50V, focusing potential = 250 V, temperature = 450°C, and detector voltage = 2300 V. LC/MS analyses were undertaken by high-resolution electrospray ionization-mass spectrometry (R ≥ 10,000; tolerance for mass accuracy = 5 ppm). As standards, (+)-catechin (Tokyo Chemical Industry, Tokyo, Japan) and (–)-epicatechin (Sigma-Aldrich) were used.

### Scavenging effects on the stable radical DPPH

GSE and Tx were dissolved in pure water to be designated concentrations followed by filtration (pore size, 0.22 μm). An aliquot (80 μL) of each aqueous solution was mixed with 16 μL of 100 mM Tris-HCl buffer (pH 7.5), 64 μL of 100% ethanol, and 40 μL of 1 mM DPPH dissolved in 100% ethanol in a well of 96-well microplate. The plate was then left in a light-shielding place for 20 min. Absorbance at 520 nm was read by the microplate reader (FilterMax F5). Rate of DPPH scavenging was calculated according to the following equation:

((A520in the solvent control–A520in specimen)/A520in the solvent control)×100,

where A520 is absorbance at 520 nm.

### Scavenging effect on the superoxide anion radical (O_2_^–•^)

GSE and Tx were dissolved in pure water to be designated concentrations followed by filtration (pore size, 0.22 μm). Scavenging activity for O_2_^–•^ was determined by the ESR-spin trapping method, as described in our previous studies [27,28]. An aliquot (50 μL) of 2 mM HPX, 30 μL of 14 M DMSO, 50 μL of a sample, 20 μL of 4.5 M DMPO, and 50 μL of 0.4 U/mL XOD were placed in a test tube and mixed. The mixture was transferred to an ESR spectrometry cell. DMPO-OOH (a spin adduct of DMPO and O_2_^–•^) was quantified 100 s after XOD addition. TEMPOL (2 μM) was used as a standard sample to calculate the concentration of DMPO-OOH, and the ESR spectrum of the manganese ion, which was equipped in the ESR cavity, was used as an internal standard. The measurement conditions for ESR (X-band ESR Spectrometer; JES-FA-100; JEOL, Tokyo, Japan) were: field sweep, 331.92–341.92 mT; field modulation frequency, 100 kHz; field modulation width, 0.1 mT; amplitude, 200; sweep time, 2 min; time constant, 0.03 s; microwave frequency, 9.420 GHz; microwave power, 4 mW. In an experiment for kinetic analyses by double-reciprocal plots, different concentrations of DMPO were added to the reaction system, as described in our previous studies [27,29,30]. Instead of different concentrations of GSE and Tx, different concentrations of SOD (a scavenger of O_2_^–•^) or of allopurinol (an XOD inhibitor) were added to the system.

### Scavenging effect on the hydroxyl radical (^•^OH)

A non-thermal atmospheric pressure plasma-jet device was used as a ^•^OH generator. The device was connected to a sinusoidal voltage power source with a voltage of 3 kV. Helium gas at a flow rate of 3 L/min was used as a feeding gas at atmospheric pressure. Using a plasma-jet, we irradiated an aliquot (500 μL) of a reaction mixture comprising designated concentrations of test substances (GSE, Tx) and 300 mM DMPO dissolved in pure water for GSE and in phosphate buffer (PB; pH 7.4) for Tx. Tx was dissolved in PB because it could not be dissolved in pure water at 1.0 mg/mL. Each mixture was transferred to an ESR spectrometry cell and the DMPO–OH spin adduct quantified 30 s after irradiation. DMPO-OH concentration was calculated in a similar way to that for O_2_^–•^ determination except that 5 μM TEMPOL was used as a standard. Measurement conditions for ESR (X-band ESR Spectrometer; JES-FA-100) were identical to those described for O_2_^–•^ determination.

### Scavenging effect on singlet oxygen (^1^O_2_)

^1^O_2_ was generated by irradiation using laser light, as described in our previous studies [31,32]. Output power of the laser was set at 40 mW. When a semi-micro cuvette containing 200 μL of sample was set in the experimental device, the area of the sample irradiated by the laser was approximately 5 × 5 mm, resulting in an energy dose of 160 mW/cm^2^. The light path of the cuvette was 10 mm. In the experiment, NaN_3_ (a specific quencher of ^1^O_2_) was used as a positive control.

A reaction mixture was prepared to contain 50 mM TPC, designated concentrations of test substances (GSE, Tx, NaN_3_), and 10 μM rose bengal in PB. Immediately after mixing, the cuvette was set in the experimental laser device. The sample in the cuvette was irradiated by laser light for 60 s. After laser irradiation, the sample was transferred to a quartz cell and the ESR spectrum was recorded on an X-band ESR Spectrometer (JES-FA-100). Measurement conditions for the ESR were: field sweep, 330.50–340.50 mT; field modulation frequency, 100 kHz; field modulation width, 0.05 mT; amplitude, 200; sweep time, 2 min; time constant, 0.03 s; microwave frequency, 9.420 GHz; microwave power, 4 mW. To calculate the spin concentration of the nitroxide radical generated through TPC oxidation by ^1^O_2_, 20 μM TEMPOL was used as a standard and the ESR spectrum of the manganese ion, which was equipped in the ESR cavity was used as an internal standard.

To ascertain if test substances reacted with the nitroxide radical, a reaction mixture containing 50 mM TPC and 10 μM rose bengal was irradiated with laser light for 60 s followed by addition of GSE or Tx (final concentration, 1 mg/mL). The ESR spectrum was recorded on an X-band ESR Spectrometer (JES-FA-100), as described above.

### Scavenging effect on H_2_O_2_

GSE and Tx were dissolved in 10 μM H_2_O_2_ to be designated concentrations. H_2_O_2_ concentration was determined by the colorimetric method based on the peroxide-mediated oxidation of Fe^2+^ followed by the reaction of Fe^3+^ with xylenol orange [33].

### Cell culture

hGFs were purchased from Primary Cell (Sapporo, Japan). Dulbecco’s modified Eagle’s medium (DMEM; Thermo Fisher Scientific, Waltham, MA, USA) containing 10% fetal bovine serum (FBS; Thermo Fisher Scientific), 100 U/mL penicillin (Wako Pure Chemicals Industries), and 0.1 mg/mL streptomycin (Wako Pure Chemicals Industries) were used as a medium for cell culture. An aliquot (100 μL) of the cell suspension (2 × 10^4^ cells/mL) was placed in each well of a 96-well culture plate. In experiments in which an intracellular ROS assay was conducted, a black 96-well culture plate was used. Plates were incubated at 37°C in a humidified atmosphere of 5% CO_2_ for 4–6 days for 100% confluence.

### Exposure of cells to H_2_O_2_, and determination of intracellular ROS and cell viability

The intracellular formation of ROS induced by H_2_O_2_ was determined using 5-(and-6)-chloromethyl-2′,7′-dichlorodihydrofluorescein diacetate, acetyl ester (CM-H2DCFDA; Life Technologies, Eugene, OR, USA) for an intracellular ROS assay [34]. After washing cells twice with phosphate-buffered saline (PBS; pH 7.4), 100 μL of 10 μM CM-H2DCFDA dissolved in serum-free DMEM was added to each well followed by incubation at 37°C in a humidified atmosphere of 5% CO_2_ for 1 h. After washing twice with PBS, cells were exposed to 0.063 mg/mL and 0.25 mg/mL of GSE or of Tx dissolved in sterile physiologic (0.9%) saline for 1 min. After washing twice with PBS, 100 μL of 10 mM H_2_O_2_ prepared in serum-free DMEM was added to each well and incubated for 20 min. Fluorescence was read at excitation and emission wavelengths of 485 and 535 nm, respectively, using the microplate reader (FilterMax F5).

Cell viability was determined by the methyl thiazolyl tetrazolium (MTT) assay [35,36], in which insoluble formazan converted from MTT was quantified at 595 nm by colorimetric means using a microplate reader (FilterMax F5). The MTT assay was carried out using a TACS MTT Cell Proliferation Assay kit (Trevigen, Gaithersburg, MD, USA). Similar to the ROS assay, cells were treated with GSE and Tx for 1 min followed by 10 mM H_2_O_2_-load for 20 min. After washing twice with DMEM supplemented with 10% FBS, cells were incubated for a further 24 h and the MTT assay was conducted to measure cell viability.

### Exposure of cells to acid-electrolyzed water (AEW), and determination of intracellular ROS and cell viability

NaCl solution (0.08% *w/v*) was electrolyzed for 15 min using a batch-type Electrolyzed Water Generator (Altron Mini AL-700A; Altec, Nagano, Japan) at a regular AC voltage of 100 V and a rated current of 0.6 A. Characteristic values of the resultant AEW were determined using a pH/ORP Meter (SG2; Mettler-Toledo, Columbus, OH, USA) for pH and oxidation-redox potential (ORP), and a Residual Chloride Meter (HI196771C; Hanna Instruments Japan, Tokyo, Japan) for residual chloride concentrations. pH, ORP, and residual chloride concentration of undiluted AEW were 2.4, 1176 mV, and 58 ppm, respectively.

After washing cells twice with PBS, cells were treated with 0.063 and 0.25 mg/mL of GSE or Tx dissolved in sterile physiologic saline for 1 min. Cells were then exposed to AEW for 30 s before washing with PBS and incubation for a further 1 h in serum-free DMEM containing 10 μM CM-H2DCFDA. After incubation, fluorescence was read at excitation and emission wavelengths of 485 and 535 nm, respectively, using the microplate reader (FilterMax F5). Since the treatment time of AEW was as short as 30 s because of its extremely cytotoxic effect [37], it was thought that 30 s was too short for DCFH (2’, 7’-dichlorodihydrofluorescin), a product deacetylated by cellular esterase, to react with ROS derived from AEW. In addition, our previous study revealed that intracellular ROS was formed even after AEW exposure [37]. Hence, in this assay, CM-H2DCFDA was post-loaded. Similar to the ROS assay, cells were treated with GSE and Tx for 1 min, followed by exposure to AEW for 30 s. After washing twice with DMEM supplemented with 10% FBS, cells were incubated for a further 24 h. An MTT assay was conducted to measure cell viability.

### Exposure of cells to ^1^O_2_, and determination of cell viability

^1^O_2_ was generated by laser-light irradiation using rose bengal at 532 nm, as described above. We used an experimental laser device equipped with the second harmonic of the Nd-YAG laser (PAX, Sendai, Japan). Output power of the laser was set at 40 mW. The diameter of the irradiation field was set to be equal to that of the well (6.4 mm) so that almost all of the light could pass through the test solution. Thus, the energy density was calculated to be 124 mW/cm^2^. A reaction mixture was prepared to contain designated concentrations of test substances (GSE, Tx), and 10 μM rose bengal in PB.

After washing cells twice with PBS, 100 μL of the reaction mixture was added to each well followed by laser-light irradiation for 1 min. Immediately after irradiation, cells were washed twice with DMEM supplemented with 10% FBS, and incubated for a further 24 h to determine cell viability by the MTT assay.

### Exposure of cells to pure water, and determination of intracellular ROS and cell viability

After washing cells twice with PBS, 100 μL of 10 μM CM-H2DCFDA was loaded for 1 h, as described above. After washing twice with PBS, cells were exposed to 0.063 and 0.25 mg/mL of GSE or Tx dissolved in sterile physiologic saline for 1 min. After washing twice with PBS, 100 μL of pure water was added to each well, and incubated for 5 min followed by washing with serum-free DMEM. The measurement of intracellular ROS was determined as described above. Similar to the ROS assay, cells were treated with GSE and Tx for 1 min followed by exposure to pure water for 5 min. Immediately after washing twice with DMEM supplemented with 10% FBS, we conducted an MTT assay to ascertain cell viability.

### Statistical analyses

Statistical analyses were undertaken using the Tukey–Kramer multiple comparison test for pairwise comparisons. P<0.05 was considered significant.

## Results

### Polyphenol assay and LC/MS analyses of GSE

**Fig 1:**
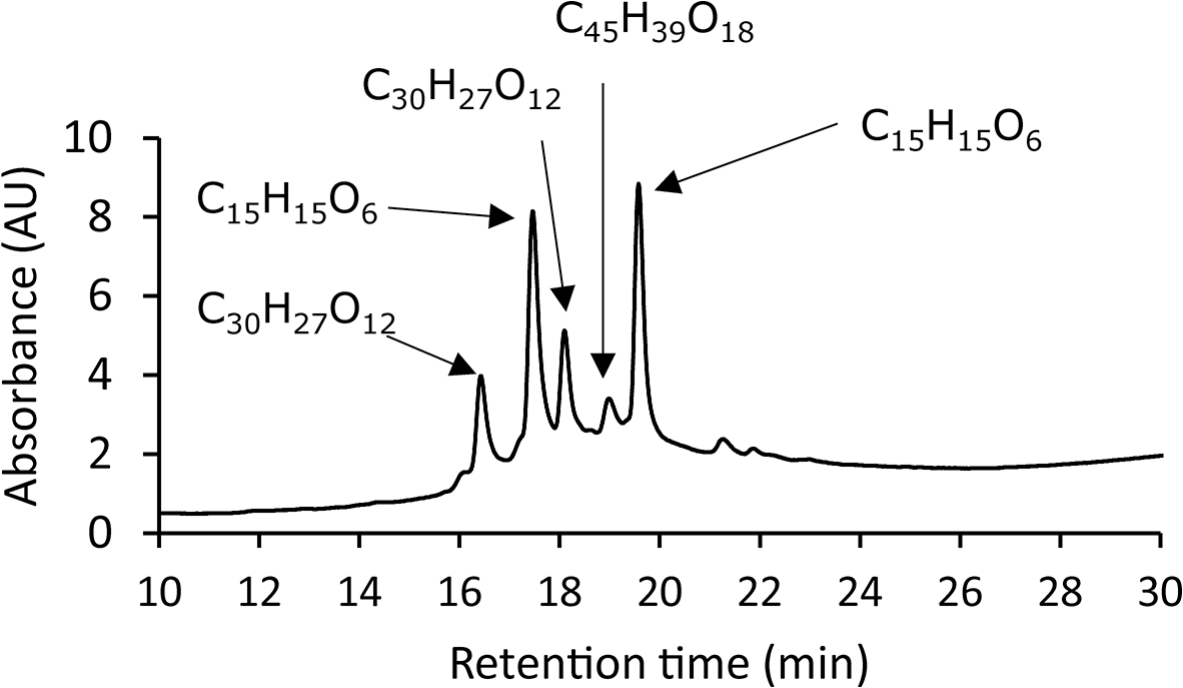
Representative LC chromatogram of GSE solution.

**Fig 2:**
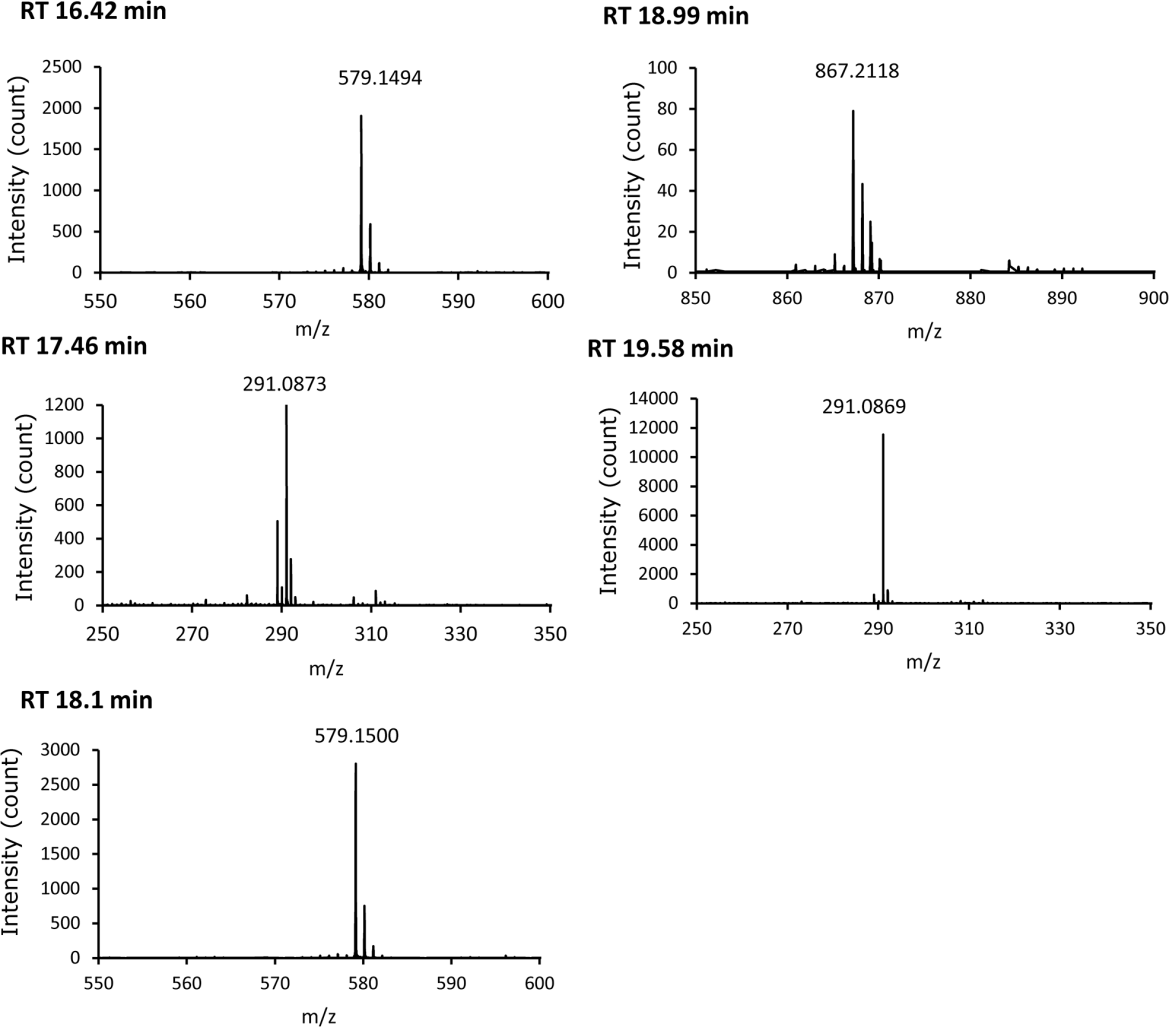
Mass spectra of the peaks obtained at the designated retention times.

Total polyphenol content in GSE expressed as gallic-acid equivalence was 84% (*wt/wt*). According to the manufacturer of GSE used in the present study (Indena), GSE comprised (+)-catechin, (–)-epicatechin, and catechin oligomers. We undertook LC/MS analyses based on this information. Results of LC/MS analyses are summarized in Table 1. A representative LC chromatogram and mass spectra of the peaks obtained at retention times of 16.42, 17.46, 18.10, 18.99, and 19.58 min are shown in Fig 1 and Fig 2, respectively.

**Table 1:** LC/MS analysis.

Sample	Retention time (min)	Molecular formula	Calculated m/z	Found m/z	ppm error	Concentration
(+)-catechin	17.49	C_15_H_15_O_6_	291.0863	291.0872	3.0408	
(-)-epicatechin	19.53	C_15_H_15_O_6_	291.0863	291.086	-1.0816	
GSE	16.42	C_30_H_27_O_12_	579.1497	579.1494	-0.5234	
	17.46	C_15_H_15_O_6_	291.0863	291.0873	3.3843	0.121 mg/mL
	18.1	C_30_H_27_O_12_	579.1497	579.15	3.9658	
	18.99	C_45_H_39_O_18_	867.213	867.2118	-1.4892	
	19.58	C_15_H_15_O_6_	291.0863	291.0869	2.0101	0.066 mg/mL

The ESI mass spectra clearly showed that (+)-catechin (calcd. for C_15_H_15_O_6_, 291.0863), (–)-epicatechin (calcd. for C_15_H_15_O_6_, 291.0863), catechin dimer (calcd. for C_30_H_27_O_12_, 579.1494), and catechin trimer (calcd. for C_45_H_39_O_18_, 867.213) were contained in GSE. The calculated concentrations of (+)-catechin and (–)-epicatechin were 12.1% (*wt/wt*) and 6.6% (*wt/wt*), respectively.

### Antioxidant properties of GSE *in vitro*

**Fig 3:**
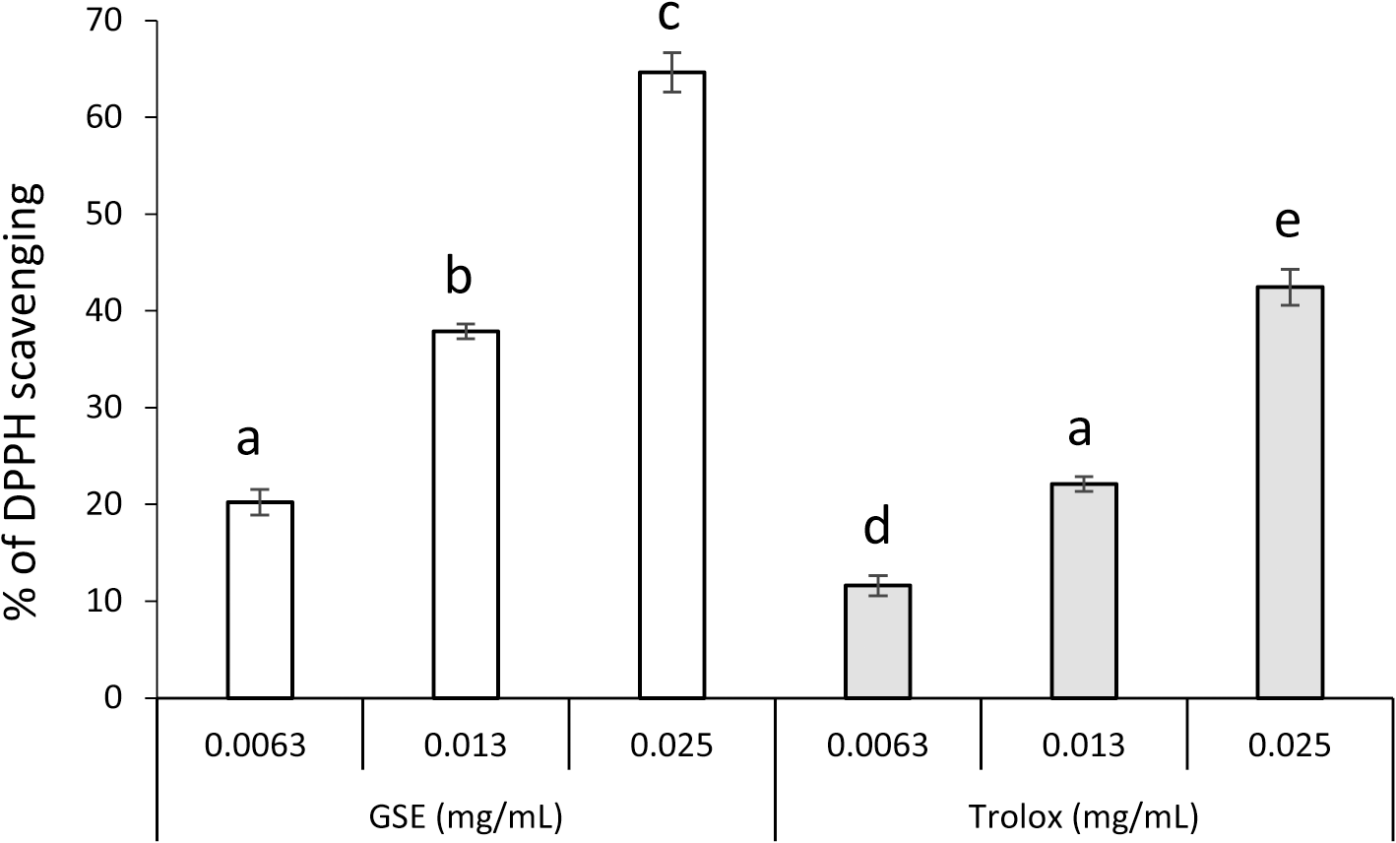
Scavenging activity of GSE upon DPPH. Each value is the mean ± standard deviation (n = 3). Significant differences (p < 0.01) within each group are denoted by different letters (*i*.*e*., bars with the same letter are not significantly different).

The scavenging effect of GSE on DPPH is shown in Fig 3. GSE and Tx scavenged DPPH in a concentration-dependent manner. The effect of GSE was slightly more potent than that of Tx because the effects of 0.0063 and 0.013 mg/mL of GSE were comparable with those of 0.013 and 0.025 mg/mL of Tx, respectively.

**Fig 4:**
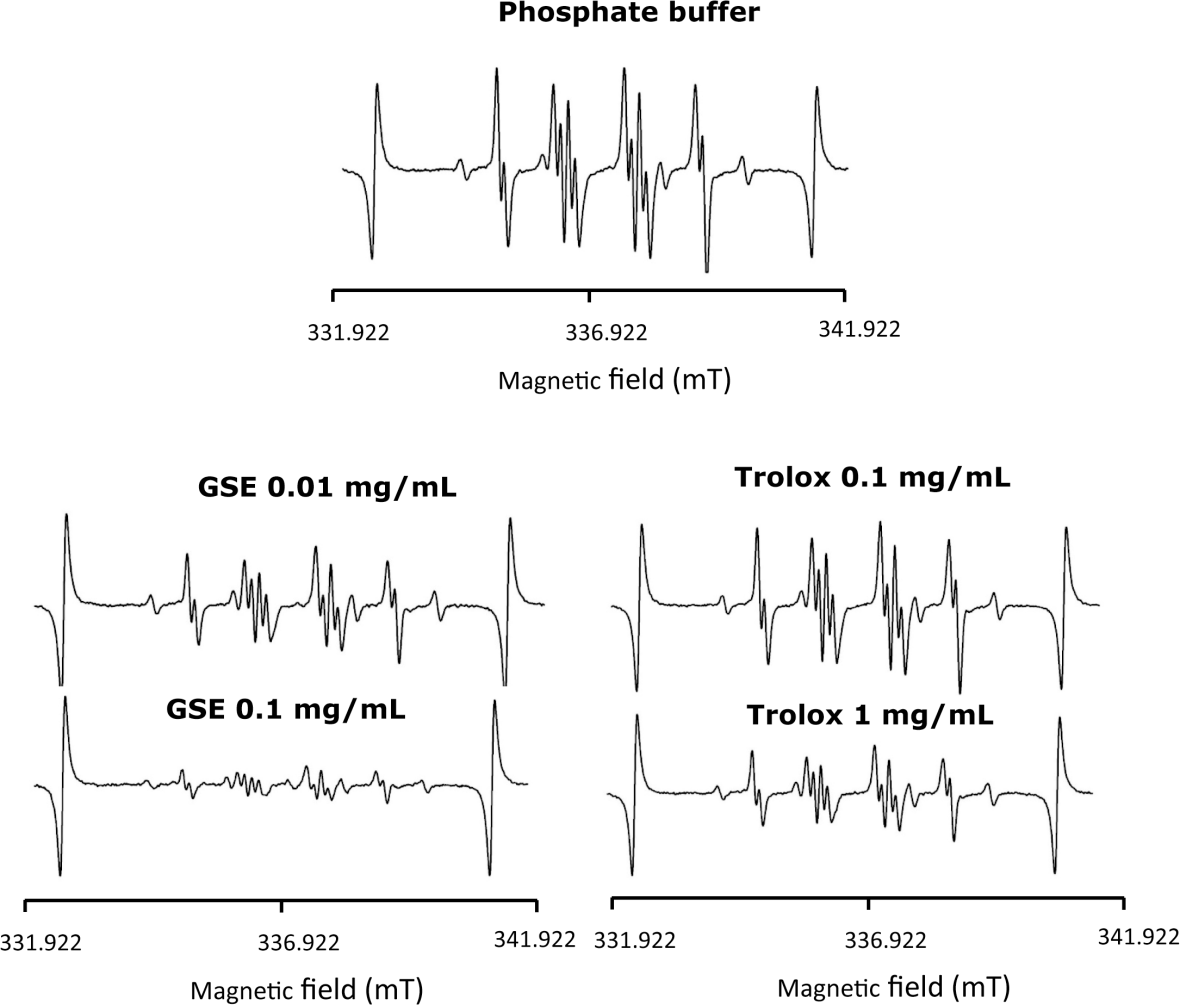
Representative ESR spectra obtained from the HPX-XOD reaction in the presence of GSE and Tx.

**Fig 5:**
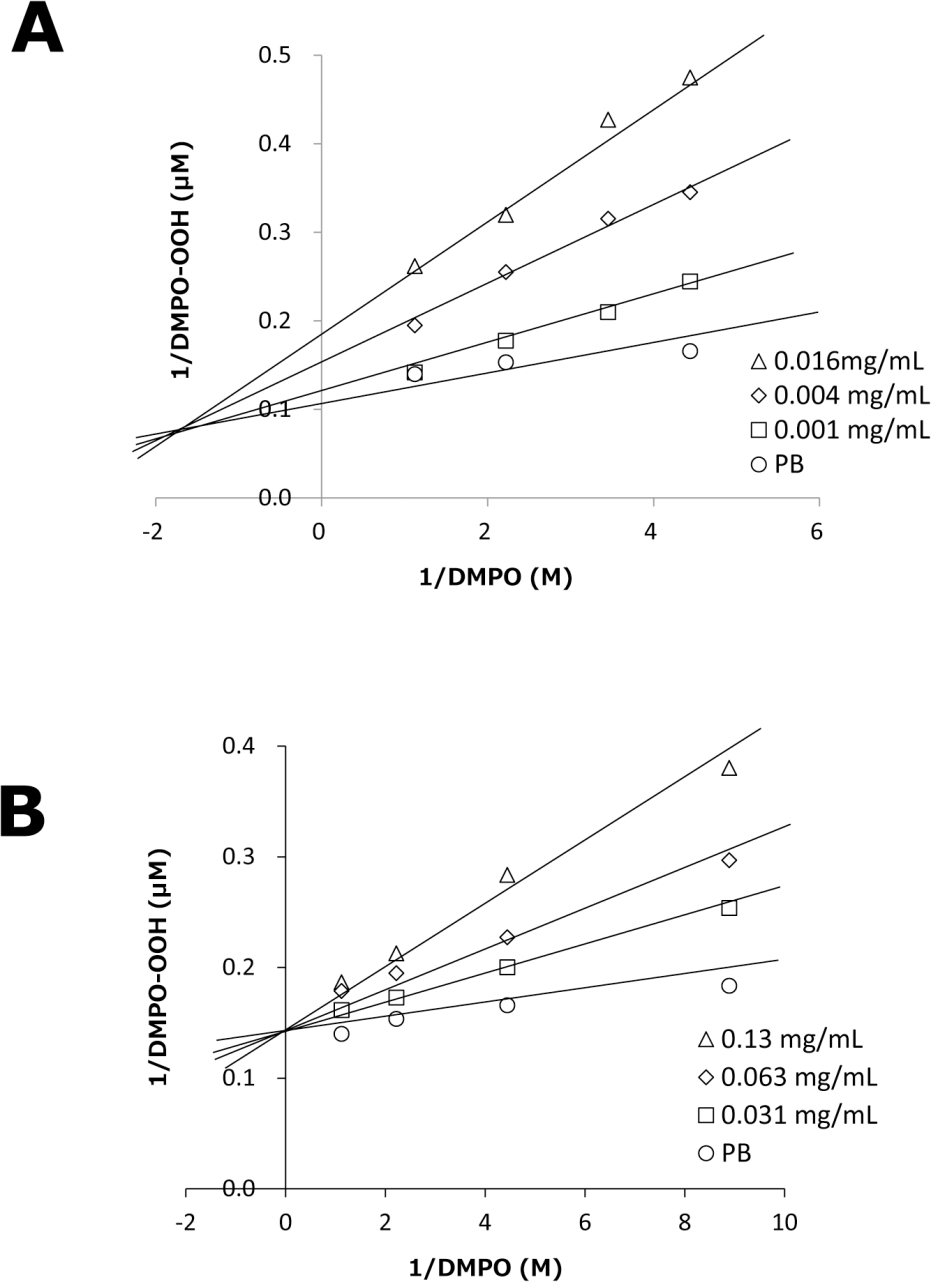
Double-reciprocal plots of formation of DMPO-OH *vs*. DMPO concentrations. The plots were obtained at changing fixed concentration of GSE (A) and Tx (B). Each value is the mean of duplicate determinations.

The scavenging effect of GSE on O_2_^–•^ generated by the HPX-XOD reaction was examined by ESR-spin trapping. When a spin trapping agent, DMPO, was added to a solution of the HPX-XOD reaction system, an ESR signal with hyperfine coupling constants of aN = 1.37 mT, aHβ = 1.10 mT, and aHγ = 0.12 mT was observed. This signal was assigned to DMPO-OOH (spin adduct of DMPO and O_2_^−•^) by the hyperfine coupling constants [38]. Fig 4 shows the representative ESR spectra of solvent control as well as different concentrations of GSE and Tx. The signal intensity of DMPO-OOH was clearly reduced by GSE and Tx in a concentration-dependent manner, and the magnitude of the reduction by GSE was much greater than that by Tx when compared with the concentrations needed to reduce the yield of DMPO-OOH. The reduction of the signal intensity of DMPO-OOH is reflected by the ability to scavenge O_2_^−•^ and/or to interfere with the HPX-XOD reaction [27, 29]. Thus, to ascertain if test substances interfere with the enzyme reaction of HPX-XOD, the ESR spin-trapping method was used to evaluate the competitive reaction between DMPO and samples or reference agents. Fig 5 shows double-reciprocal plots (corresponding to Lineweaver–Burk plots as in the kinetics of enzyme actions) for GSE and Tx. In the case of SOD, a linear pattern with an intersection on the y axis that denotes competitive scavenging for O_2_^−•^ with DMPO was obtained (data not shown). In the case of the XOD inhibitor allopurinol [39], a parallel linear pattern that denotes interference with the HPX-XOD reaction was obtained (data not shown). In the case of Tx, the linear and intersecting patterns of the double-reciprocal plot were similar to those of SOD, suggesting that inhibition of DMPO-OOH formation by Tx was attributable to a scavenging effect on O_2_^−•^. In the case of GSE, a linear pattern with an intersection shifted to the negative side of the x axis was observed, suggesting that DMPO-OOH formation was inhibited not only by scavenging of O_2_^−•^ but also by interference with the HPX-XOD reaction (“mixed reaction”).

**Fig 6:**
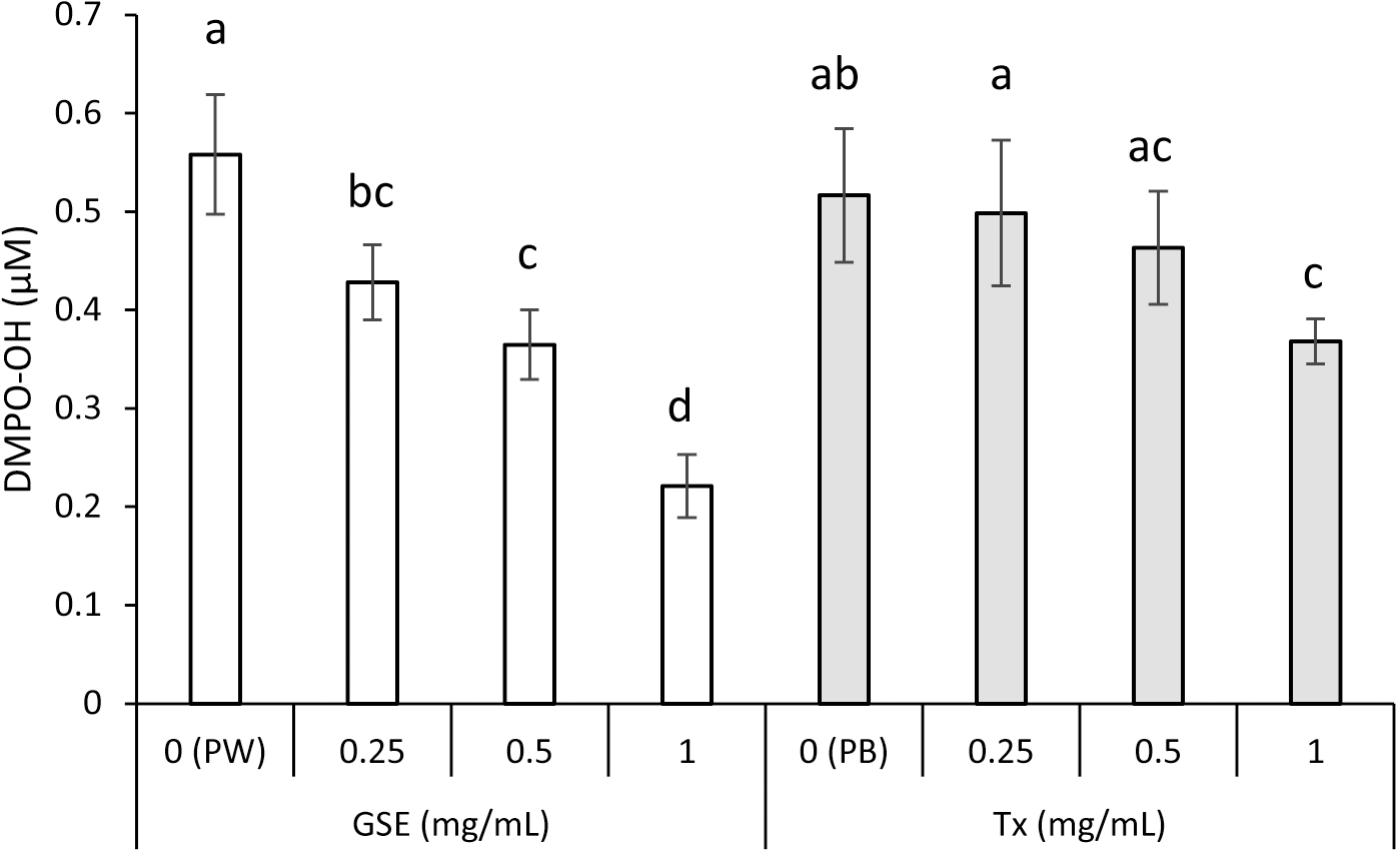
Scavenging activity of GSE upon ^•^OH generated by plasma-jet irradiation of an aqueous solution. Each value is the mean ± standard deviation (n = 4). Significant differences (p < 0.05) within each group are denoted by different letters (*i*.*e*., bars with the same letter are not significantly different). PW and PB stand for pure water and phosphate buffer used as solvents for GSE and Tx, respectively.

The scavenging effects of GSE on ^•^OH generated by the plasma-jet irradiation of water or PB were examined. When DMPO was added to a solution, an ESR signal with hyperfine coupling constants of aN = 1.49 and aH = 1.49 mT was observed. This signal was assigned to DMPO-OH (spin adduct of DMPO and ^•^OH) by the hyperfine coupling constants [38]. Signal intensity of DMPO-OH probably reflects an ability to scavenge ^•^OH because when dimethyl sulfoxide (DMSO; an authentic scavenger of ^•^OH) was added to a mixture, a signal of DMPO-CR (adduct of a carbon-center radical derived from DMSO and ^•^OH) appeared concomitantly with disappearance of the DMPO-OH signal (data not shown), suggesting that free ^•^OH was generated by plasma-jet irradiation. Fig 6 summarizes the suppressive effect of GSE on ^•^OH yield expressed as DMPO-OH. GSE and Tx suppressed ^•^OH yield significantly (p<0.01) in a concentration-dependent manner. Also, the effect of GSE was slightly more potent than that of Tx because the effects of 0.25 and 0.5 mg/mL of GSE were comparable with those of 0.5 and 1.0 mg/mL of Tx, respectively.

**Fig 7:**
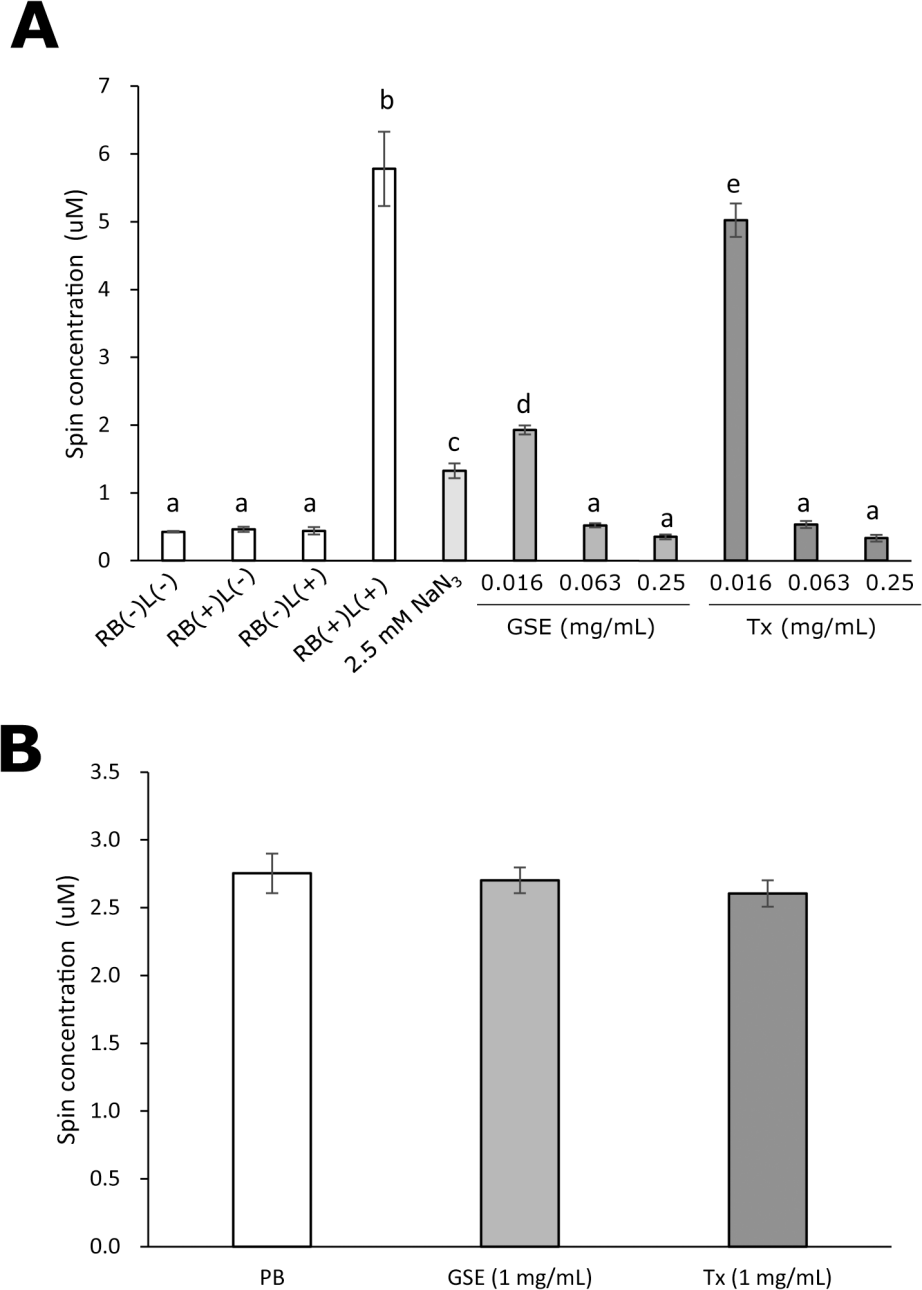
Scavenging activity of GSE upon photo-generated ^1^O_2_. Each value is the mean ± standard deviation (n = 3). Significant differences (p < 0.01) within each group are denoted by different letters (*i*.*e*., bars with the same letter are not significantly different).

The scavenging effect of GSE on photo-generated ^1^O_2_ was determined by ESR analyses. The calculated spin concentrations of the nitroxide radical derived from TPC oxidation by ^1^O_2_ are summarized in Fig 7A, in which RB(–)L(–), RB(+)L(–), RB(–)L(+), and RB(+)L(+) indicate no treatment, treatment with 10 μM rose bengal alone, treatment with laser-light irradiation alone, and laser-light irradiation of 10 μM rose bengal, respectively. Under conditions of RB(–)L(–), RB(+)L(–), and RB(–)L(+), yields of the nitroxide radical were very low whereas, under the condition of RB(+)L(+), the yield of the radical increased prominently. Similar to the scavenging effect on DPPH and ^•^OH, the increased yield of the nitroxide radical by photo-irradiated rose bengal was clearly reduced by GSE and Tx in a concentration-dependent manner. Also, the magnitude of the reduction by GSE was relatively greater than that by Tx when compared with the concentrations needed to reduce the yield of the nitroxide radical. NaN_3_ (2.5 mM) used as a positive control also prominently reduced the yield of the radical. To confirm that neither GSE nor Tx reacts with the nitroxide radical, the effect of post-treatment with GSE and Tx on the yield of the nitroxide radical generated by photo-irradiation of a reaction mixture containing 50 mM TPC and 10 μM rose bengal was examined. The calculated spin concentrations are summarized in Fig 7B, and showed that levels of the nitroxide radical were not changed significantly by post-treatment with GSE and Tx.

**Fig 8:**
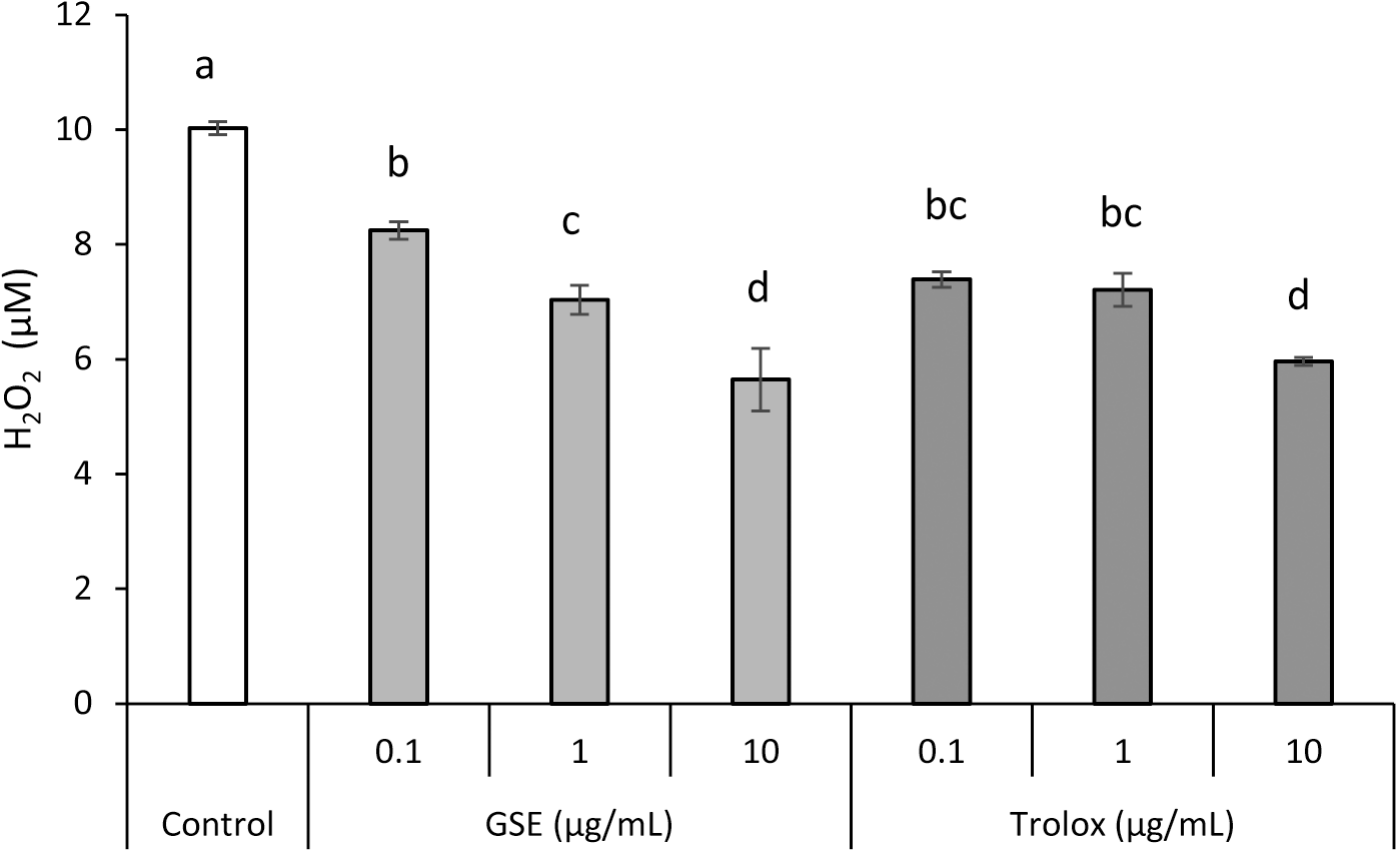
Scavenging action of GSE upon H_2_O_2_. Each value is the mean ± standard deviation (n = 3). Significant differences (p < 0.01) within each group are denoted by different letters (*i*.*e*., bars with the same letter are not significantly different).

The scavenging effect of GSE on H_2_O_2_ is summarized in Fig 8. GSE and Tx scavenged H_2_O_2_ in a concentration-dependent manner.

### Cytoprotective effects of GSE on hGFs exposed to oxidative stressors

**Fig 9:**
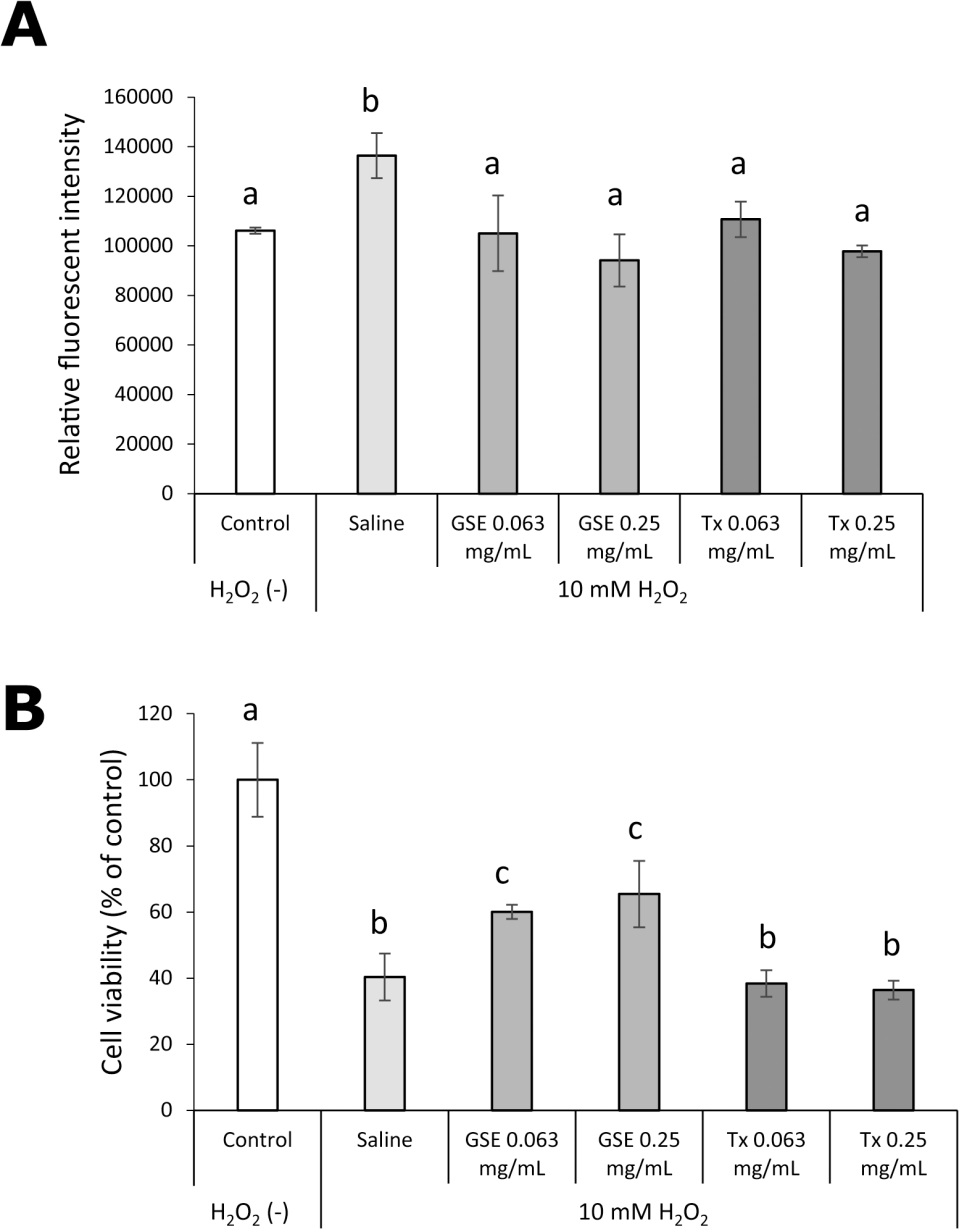
Effect of GSE on hGFs exposed to H_2_O_2_. Intracellular formation of ROS (A) in, and the viability (B) of hGFs were examined. Each value is the mean ± standard deviation (n = 4). Significant differences (p < 0.01) within each group are denoted by different letters (*i*.*e*., bars with the same letter are not significantly different).

The effect of GSE pretreatment for 1 min on H_2_O_2_-induced oxidative stress and cytotoxicity in hGFs is shown in Fig 9. The increased intracellular formation of ROS upon exposure to H_2_O_2_ was suppressed significantly by pretreatment with 0.63 and 0.25 mg/mL of GSE, and the suppressive effect of GSE was comparable with that of Tx. A decrease in survival of viable cells 24 h after exposure to H_2_O_2_ was also prevented significantly (p<0.01) by GSE pretreatment, but Tx pretreatment failed to protect hGFs from the toxic effects of H_2_O_2_.

**Fig 10:**
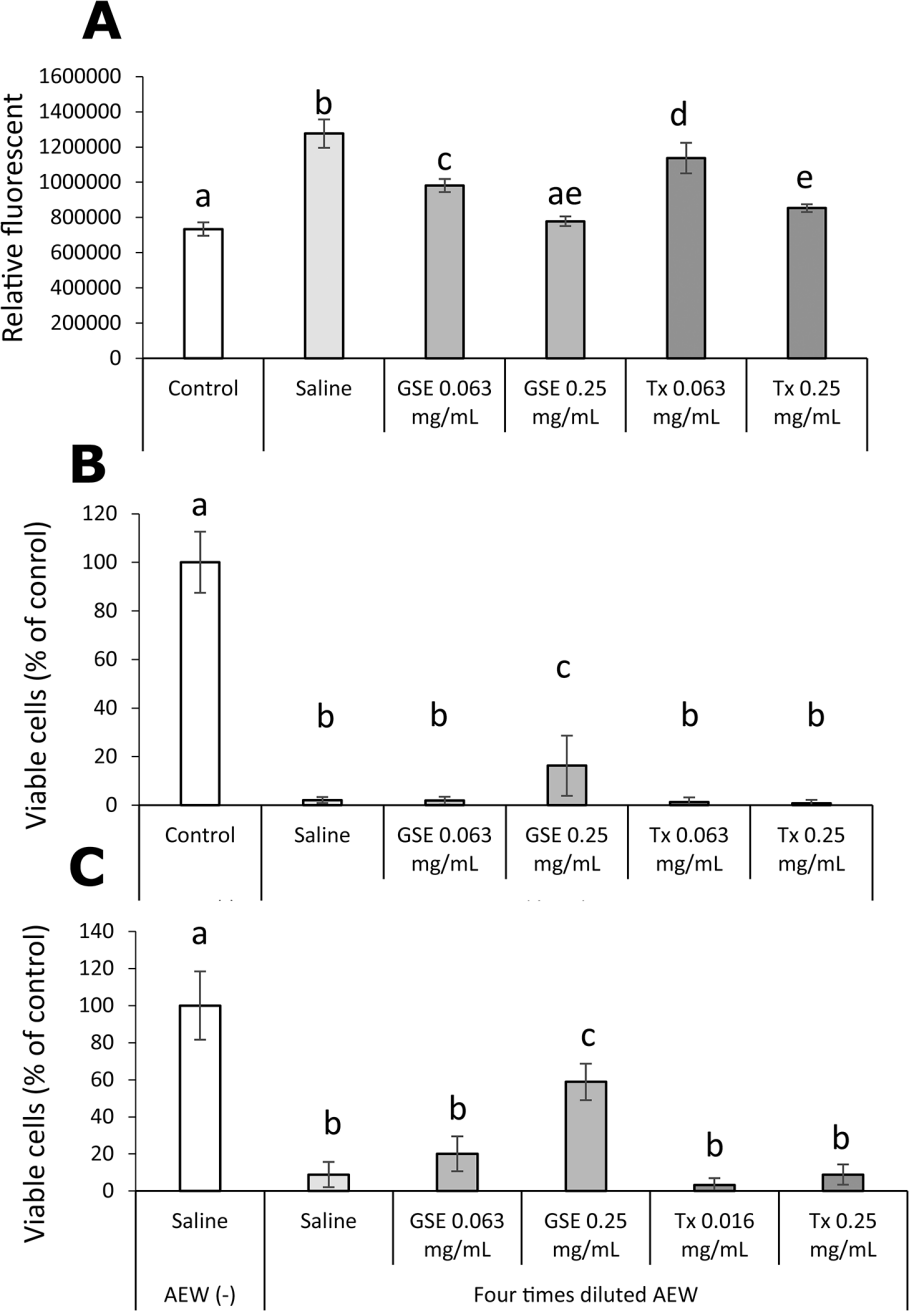
Effect of GSE on hGFs exposed to acid electrolyzed water (AEW). Intracellular formation of ROS (A) in hGFs exposed to undiluted AEW, and the viability of hGFs exposed to undiluted AEW (B) and fourfold diluted AEW (C) were examined. Each value is the mean ± standard deviation (n = 4). Significant differences (p < 0.01) within each group are denoted by different letters (*i*.*e*., bars with the same letter are not significantly different).

The effect of pretreatment with GSE on AEW-induced oxidative stress and cytotoxicity in hGFs is shown in Fig 10. Similar to the result of the H_2_O_2_ experiment, an increase in intracellular ROS induced by AEW was suppressed significantly (p<0.01) by pretreatment with not only GSE but also Tx. Cytotoxicity induced by undiluted and fourfold-diluted AEW was reduced by pretreatment with GSE but not with Tx.

**Fig 11:**
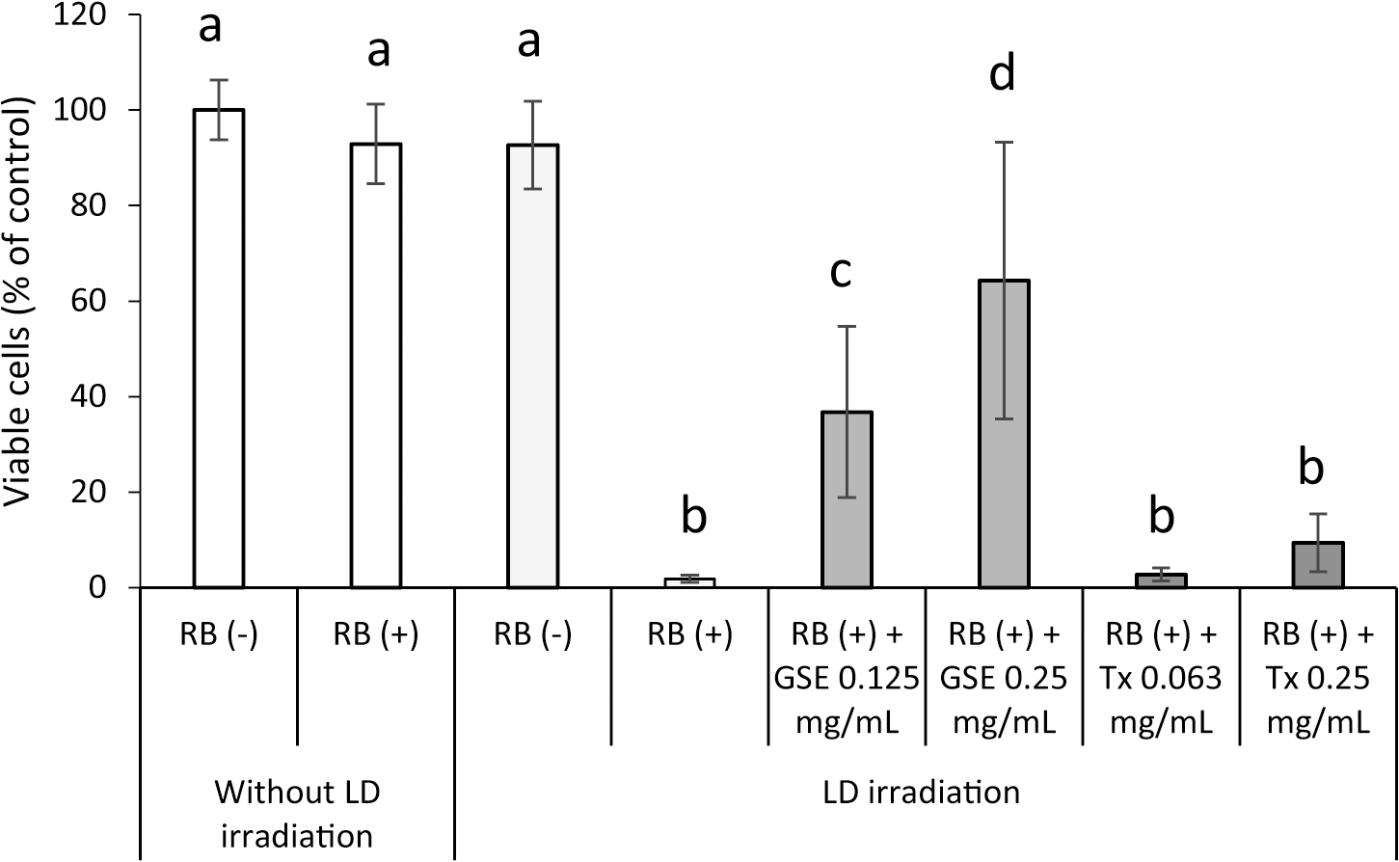
Effect of GSE on the viability of hGFs exposed to photo-generated ^1^O_2_. Each value is the mean ± standard deviation (n = 5). Significant differences (p < 0.01) within each group are denoted by different letters (*i*.*e*., bars with the same letter are not significantly different).

The effect of concomitant treatment with GSE on ^1^O_2_-induced cytotoxicity in hGFs is shown in Fig 11. As with H_2_O_2_- and AEW-induced cytotoxicity, a decrease in survival of viable cells 24 h after exposure to ^1^O_2_ was suppressed significantly (p<0.01) by concomitant treatment with 0.25 mg/mL of GSE during laser-light irradiation for 1 min, but concomitant treatment with Tx was not.

### Cytoprotective effect of GSE on hGFs exposed to a low osmotic stressor (pure water)

**Fig 12:**
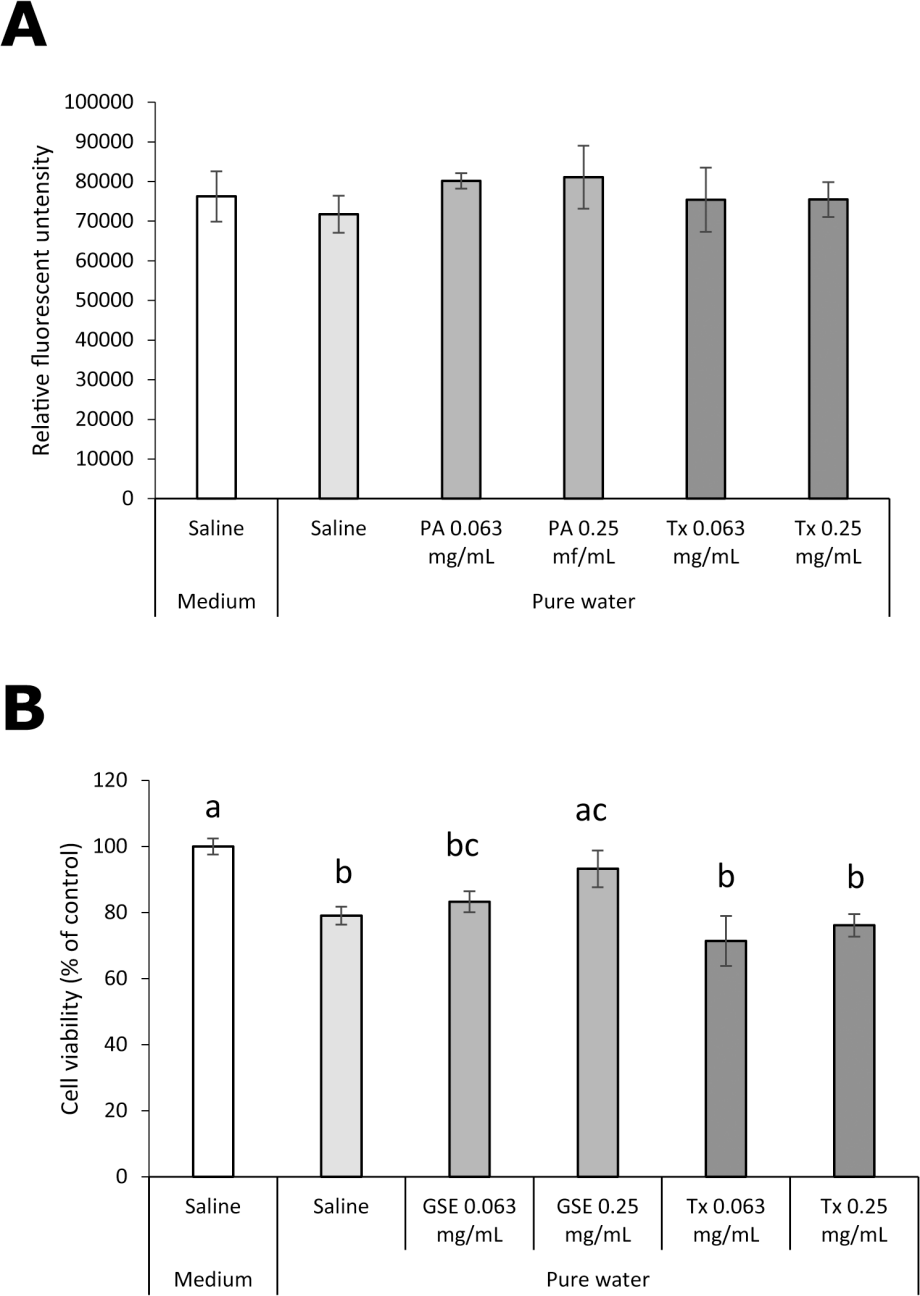
Effect of GSE on hGFs exposed to pure water. (A) Intracellular formation of ROS and (B) the viability were examined. Each value is the mean ± standard deviation (n = 4). Significant differences (p < 0.01) within each group are denoted by different letters (*i*.*e*., bars with the same letter are not significantly different).

The effect of GSE pretreatment for 1 min on intracellular ROS in and viability of hGFs exposed to pure water is shown in Fig 12. Unlike exposure to oxidative stressors, no increase in intracellular formation of ROS was found by exposure to pure water, and neither GSE nor Tx affected intracellular levels of ROS (Fig 12A). A significant decrease (p<0.01) in cell viability was found immediately after exposure to pure water (Fig 12B). Pretreatment with 0.25 mg/mL GSE significantly (p<0.01) protected hGFs from the toxic effect of exposure to pure water, whereas pretreatment with 0.063 or 0.25 mg/mL Tx did not.

## Discussion

LC/MS analyses confirmed that, as disclosed by the manufacturer (Indena), the GSE used in the present study comprised not only catechin monomers but oligomers such as proanthocyanidin. As reported in our previous study (in which short-term pretreatment with proanthocyanidin-rich GSE exerted cytoprotective effects on hGFs exposed to harsh environmental conditions), 1-min pretreatment with GSE reduced the magnitude of oxidative stress induced by H_2_O_2_ and AEW, and improved the viability of surviving cells. In the case of AEW, a very severe cytotoxic effect was induced. In our previous study, it was suggested that the cytotoxic effect of AEW is probably induced by ROS, especially ^•^OH [37]. With regard to H_2_O_2_, O_2_^-•^, ^1^O_2_, and ^•^OH, the latter is the most reactive [40,41], suggesting that intracellular ^•^OH generated *via* AEW was responsible for the severe cytotoxic effect of AEW. Conversely, although Tx (a water-soluble analog of vitamin E) attenuated oxidative stress as much as GSE, survival of viable cells was not improved, unlike the case of GSE. Similar results were obtained in the ^1^O_2_ experiment. That is, concomitant treatment with GSE during exposure of cells to ^1^O_2_ protected them from the cytotoxic effect of ^1^O_2_ but Tx treatment did not. The *in vitro* antioxidant profiles examined in the present study showed that the radical or ROS (*i*.*e*., DPPH, ^•^OH, ^1^O_2_, H_2_O_2_) scavenging activity of GSE was moderately more potent than, or similar to that of Tx. It was shown by the ESR-spin trapping method that GSE and Tx can scavenge O_2_^−•^. However, the kinetic study using analyses of double-reciprocal plots revealed that the activity between GSE and Tx could not be compared because GSE not only scavenged O_2_^−•^ directly but also interfered with the HPX-XOD reaction that was responsible for O_2_^−•^ generation. Despite the comparable *in vitro* antioxidant potential and suppressive effect of intracellular ROS in cells exposed to H_2_O_2_ and AEW between GSE and Tx, only GSE protected cells from the toxic effects of oxidative stressors, including ^1^O_2_ as well as H_2_O_2_ and AEW, in terms of survival of viable cells after exposure to stressors. Regarding a positive control, we also examined the effect of ascorbic acid on intracellular ROS in and survival of hGFs loaded with H_2_O_2_ and AEW. However, ascorbic acid neither suppressed oxidative stress nor improved cell survival, possibly due to the poor permeability of ascorbic acid into cells during such a short time as 1 min (data not shown). In other words, anti-oxidants that have an ability to exert anti-oxidative effect on cells within a short time as 1 min can only be used as a positive control.

These results suggest that the direct antioxidant potential of GSE was not a pivotal player for the cytoprotective effects expressed by the survival of viable cells because Tx did not show such cytoprotective effects. In our previous study, proanthocyanidin-rich GSE showed cytoprotective effects on hGFs in the mitotic phase exposed to low osmotic stress induced by exposure to pure water instead of medium [21], suggesting that the cytoprotective effects of GSE may be independent of its direct antioxidant action. Thus, this discrepancy in cytoprotective effects between GSE and Tx tempted us to examine further the effect on cells exposed to low osmotic stress (*i*.*e*., exposure to pure water) in relation to intracellular formation of ROS. As with exposure to H_2_O_2_ and AEW, GSE pretreatment resulted in less reduction in the viability of cells exposed to pure water, whereas Tx pretreatment showed almost no effect on cell viability. Intracellular formation of ROS was not increased in cells exposed to pure water, so the cytoprotective effects of GSE were probably exerted independently of its direct antioxidant action. The fundamental mechanism by which short-term treatment with GSE exerts cytoprotective effects on hGFs exposed to oxidative stressors should be examined further.

In conclusion, short-term treatment with GSE can protect hGFs exposed to oxidative stressors such as ROS released from inflammatory cells infiltrating gingival tissues. The antioxidant potential of GSE is unlikely to be responsible for its cytoprotective effect.
