# Correction: Cytoprotective Effects of Grape Seed Extract on Human Gingival Fibroblasts in Relation to Its Antioxidant Potential

**DOI:** 10.1371/journal.pone.0138394

**Published:** 2015-09-14

**Authors:** Yusuke Katsuda, Yoshimi Niwano, Takuji Nakashima, Takayuki Mokudai, Keisuke Nakamura, Satomi Oizumi, Taro Kanno, Hiroyasu Kanetaka, Hiroshi Egusa

Fig 11 is incorrect. The authors have provided a corrected version here.

**Fig 11:**
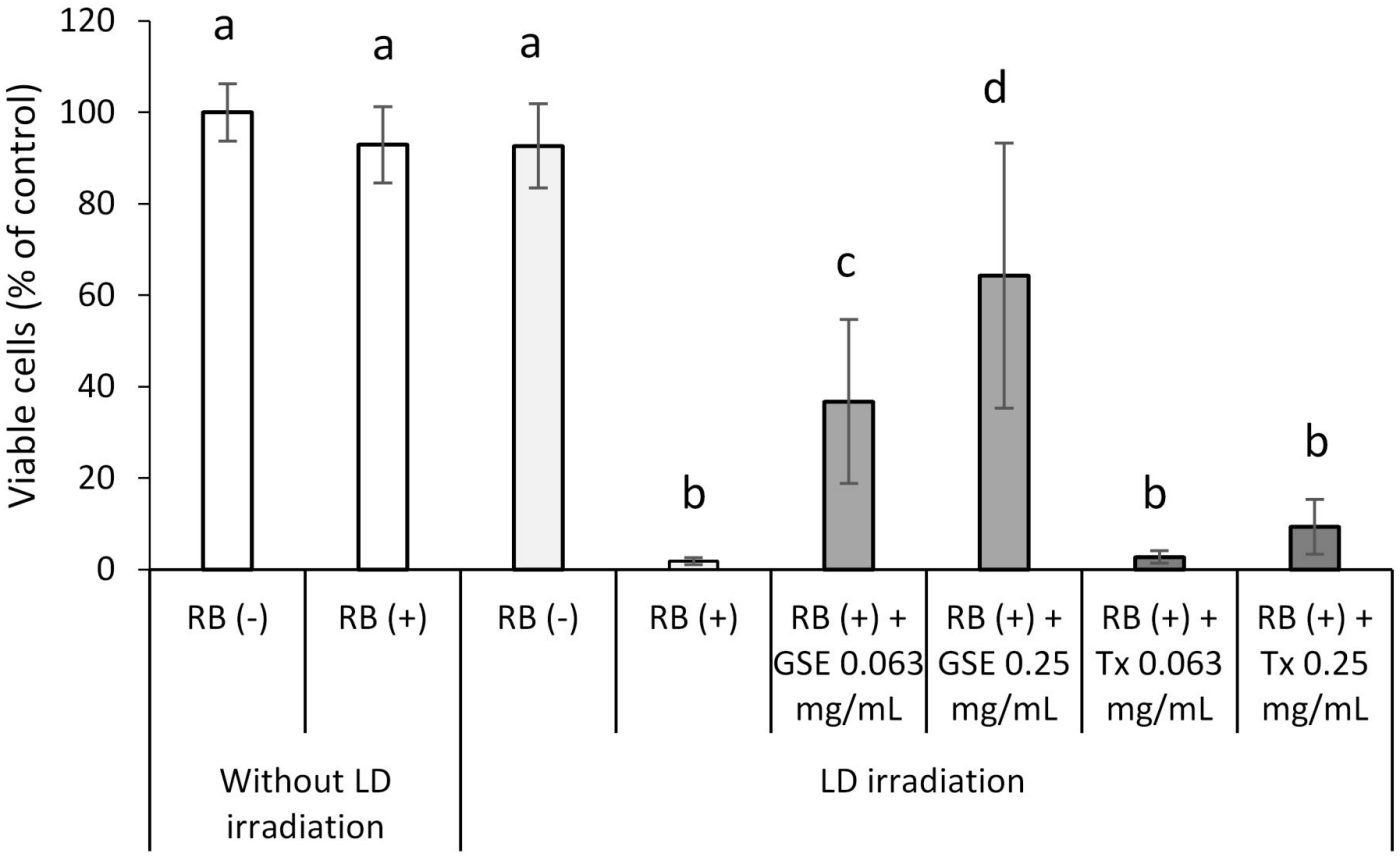
Effect of GSE on the viability of hGFs exposed to photo-generated ^1^O_2_. Each value is the mean ± standard deviation (n = 5). Significant differences (p < 0.01) within each group are denoted by different letters (*i*.*e*., bars with the same letter are not significantly different).

Fig 12 is incorrect. The authors have provided a corrected version here.

**Fig 12:**
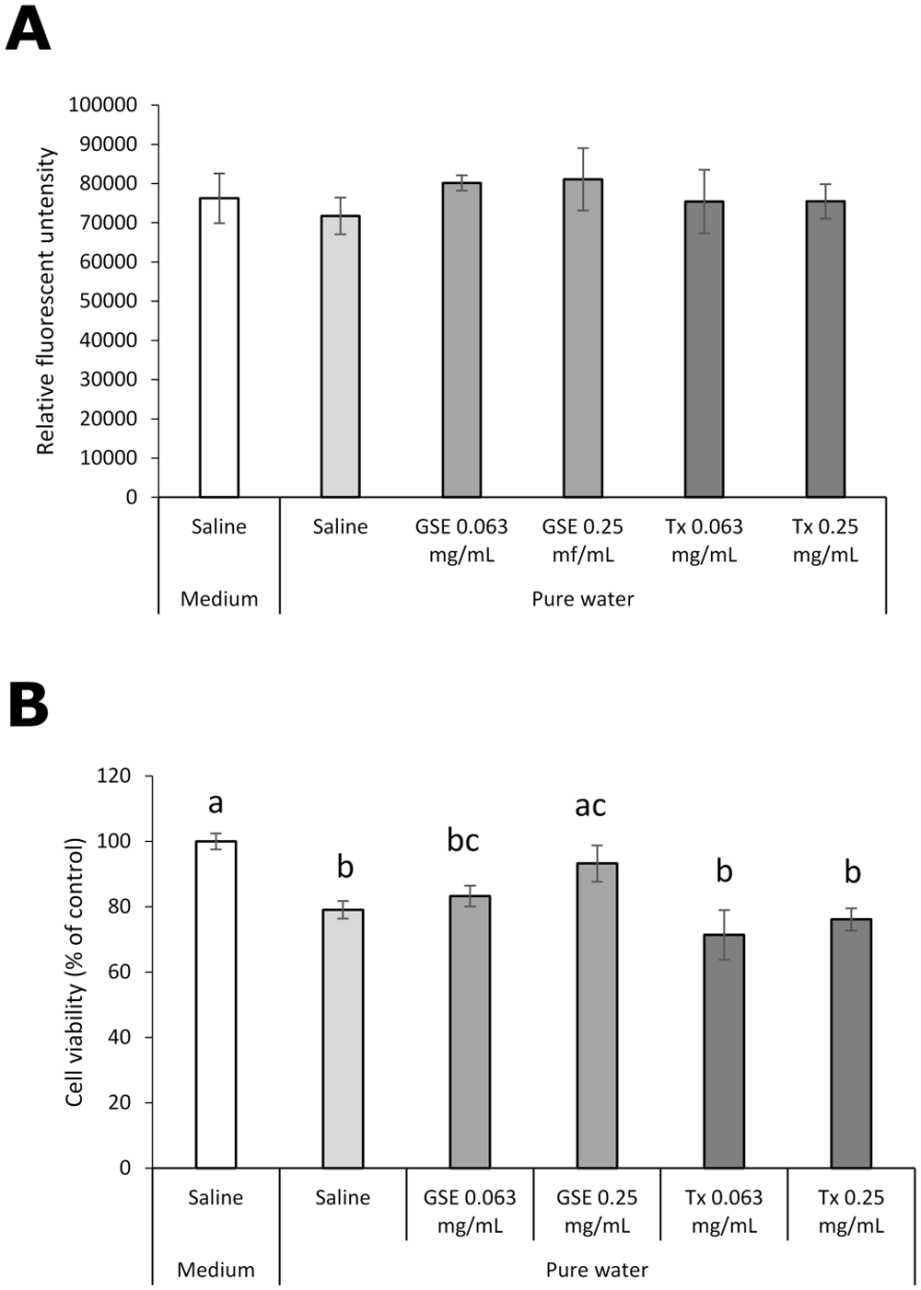
Effect of GSE on hGFs exposed to pure water. (A) Intracellular formation of ROS and (B) the viability were examined. Each value is the mean ± standard deviation (n = 4). Significant differences (p < 0.01) within each group are denoted by different letters (*i*.*e*., bars with the same letter are not significantly different).
